# Epidemiological Investigation and Virus Tracing of a Measles Outbreak in Zhoushan Islands, China, 2019

**DOI:** 10.3389/fpubh.2020.600196

**Published:** 2020-12-01

**Authors:** Hui Zhang, Can Chen, An Tang, Bing Wu, Leijie Liu, Mingyu Wu, Hongling Wang

**Affiliations:** ^1^Zhoushan Center for Disease Control and Prevention, Zhoushan, China; ^2^Putuo Center for Disease Control and Prevention, Zhoushan, China

**Keywords:** measles virus, D8 genotype, outbreak, epidemiological investigation, phylogenetic analysis

## Abstract

**Background:** Measles transmissions due to case importations challenge public health systems globally and herd immunities in all countries. In 2019, an imported measles case and its subsequently outbreak was found in the Zhoushan Islands. Here, the process of epidemiological investigation and virus tracing were summarized to provide references for the prevention and control of measles in the future.

**Materials and methods:** The data on the demographic, epidemiological, and clinical manifestation of measles cases in this outbreak were collected. The 450 bp fragments of the measles virus (MeV) N gene were amplified and sequenced. The genome of the first imported case was further isolated. Then, the maximum-likelihood and time-scaled phylogenetic analysis was conducted.

**Results:** A total of 28 measles cases were confirmed. Their onsets were between March 13 and May 18, 2019. The first patient was from the Ukraine. He was confirmed at the Fever Clinic in Zhoushan hospital on March 15, 2019 and at the same time, another patient had visited the hospital due to another illness and 10 days later, this second case had onset (March 25, 2019). The epidemic curve shows sustained community transmission. The majority of the following cases (19/26) were clustered on the Donggang street which was close to where the second case worked. The 22 measles virus strains successfully isolated from this outbreak all belonged to the D8.2a sub-cluster and clustered with the KY120864/MVs/GirSomnath.IND/42.16/[D8] which was the predominant genotype in the Ukraine during 2018-2019. The analysis of the complete D8 genotype genome pointed to the fact that this prevailing strain originated from India in 2015 and its substitution rate was estimated as 6.91 × 10^−4^ (5.64–7.98 × 10^−4^) nucleotide substitutions/site/year.

**Conclusion:** This outbreak was caused by an imported case from the Ukraine. There was a possible nosocomial infection between the first case and the second case. Then, the second case played an important role in the spread of virus due to her occupation. The molecular phylogenetic analysis could help to track the origin of the virus. Increasing and maintaining the high level of vaccination coverage (≥95%) and an efficient response to imported cases are essential to prevent and control the recurrence and outbreak of measles virus.

## Introduction

Measles is a highly contagious disease characterized by a basic reproduction number (R0) ranging from 12 to 18 (1, 2). It is caused by measles virus (MeV) mainly spreading through respiratory routes with typical symptoms of fever and rash (3). At the present time, all member states/regions of the World Health Organization (WHO) have set a goal of eliminating measles (4). This, however, has had to be revised or suspended due to a global upsurge in measles cases in 2018–2019. This includes countries who had previously verified that they had eliminated measles, where the disease has been re-established. Although China has reported record low numbers (5).

Measles is vaccine-preventable but the global elimination of measles has constantly been impacted. This is mainly due to pockets of low vaccination coverage and repeated international importations of measles cases among unvaccinated persons contacting the affected communities (6). Therefore, case base surveillance and analysis of circulating MeV strains has become important in the documentation of the interruption of measles transmission and the sources of imported cases (7). MeV was classified into eight clades including 24 genotypes that show a distinct geographic distribution published in 2012 (8). The imported measles cases always led to the geographical dispersion of measles strains and outbreaks. For example, in 2008, an outbreak of >24,300 cases in Bulgaria was caused by the imported MV/D4-Hamburg virus (9). In 2017, the imported D3 and D8 genotypes caused one of the largest measles outbreaks in Italy with 5,404 notified cases and 4,347 confirmed cases (10). In the United States, 22% of measles cases were imported and caused 66 outbreaks during the 2009–2014 period (11).

The MeV genome is 15,894 nucleotides in length and encodes a total of eight proteins (N, P, M, F, H, L, C, and V). The carboxy-terminal 450 nucleotides of N were used for measles genotyping, established by WHO (12). In China, genotype H1 has been predominantly cocirculating since the early 1990s (13), among these, several reports stated that the outbreaks were related to new imported measles virus such as the D8, B3, B4, and D11 strains (14–17). Zhoushan consists of an archipelago of islands and is the only prefecture-level city in China. The Zhoushan Islands are located at the northeast of Zhejiang Province (121°30'E~123°25'E, 29°32N~31°04'N) with ~22,200 km^2^ in land area and 1.2 million in population. Genotype H1 of measles virus was dominantly circulating in Zhoushan before 2018 (18). As a result of the special geographic features of the area, there are many ports for foreign trade and transportation. Frequent movement of a population increases the risk of infectious disease importations. In 2008, the Zhoushan Islands experienced their largest measles outbreak (115 cases) caused by imported cases in the Putuo region. In this study, we report the epidemiological investigation and virus tracing of an imported measles case and the subsequent outbreak in the Zhoushan Islands to provide our recommendations for local measles elimination and eradication in the future.

## Materials and Methods

### Case Definition, Epidemiology Investigation, and Specimen Collection

The case and outbreak definition are according to the China National Measles Surveillance Programme (19). In total, 28 patients with measles were reported by a local hospital from March to May, 2019. When the measles cases were clinically diagnosed and reported from the local hospital, the local/district (Zhoushan and Putuo) Center for Disease Control and Prevention (CDC) arrived at the scene within 24 h and then conducted an epidemiology investigation using a standardized questionnaire to collect the demographic, epidemiologic, and clinical data of these patients. The possible sources of infections, transmission routes, and close contacts were also recorded. Subsequently, staff at all levels of the medical organizations or clinics in the Zhoushan Islands were requested to react and continually seek possible measles cases based on the records of clinical surveillance. At the same time, throat swabs and serum specimens of patients were collected by the local hospital or CDC members and then transported to the Zhoushan CDC measles network lab for testing. The laboratory-confirmed patients were then isolated and treated in a specialized hospital for infectious diseases.

### Measles Virus Detection, Amplification, and Sequencing

According to the standard operational procedures (SOP) developed by China's CDC, the throat swab specimens were tested for MeV nucleic acids by RT-qPCR (Bioperfectus, China) and the serum specimens were tested for the anti-measles IgM antibody (Serion, Germany) (19). The specimens which were identified as MeV positive by RT-qPCR were kept for virus isolation and gene sequencing. The QIAamp®Viral RNA Mini kit was used to extracted the measles virus RNA according to the manufacturer's instructions (QIAGEN, Hilden, Germany). Then, the RNA was subjected to RT-PCR with specific primers to amplify the 450 bp fragment of the N gene which was used for MeV genotyping (20). The PCR products were purified and sequenced by Sangon Biotech (Shanghai, China). The complete genome of the first strain in this outbreak was further amplified and sequenced by Sangon Biotech (Shanghai, China) using Sanger dideoxy sequencing.

### Genotyping and Phylogenetic Analysis

The blast tool (https://blast.ncbi.nlm.nih.gov/Blast.cgi) was used to genotype the measles virus. The online tool MAFFT (https://maft.cbrc.jp/alignment/server) was used to align the sequences. The IQTREE v.2.0 software (21) was used to construct the maximum-likelihood (ML) phylogenetic tree of both the N gene sequences and the complete genome of the D8 genotype strains with K3P and TIM+F+R3 nucleotide substitutions models which were best-ft models, respectively in the ModerFinder software according to the Bayesian information criterion (BIC). A temporal signal in the dataset was evaluated by a root-to-tip regression based on ML phylogeny inferred using the IQTREE v.2.0 software via the program TempEst v.1.5 (22). The BEAST v.1.8.2 software (23) was used to construct the time-scaled phylogenetic tree in order to estimate the time of the most recent common ancestor (TMRCA). The appropriate molecular clock and tree models were then determined by path-sampling/stepping sampling (PS/SS) and then the exponential growth-relaxed molecular clock, GTR+G+I+F4 substitution, and constant size coalescent model were chosen, with a single run of a Markov Chain Monte Carlo (MCMC) sample chain for 100 million steps and sampling every 10,000 steps. The MCMC analysis was run for more than twice as much, and then two of them were selected to check for convergence (24). After discarding the first 10% of the chain, the effective sample size (ESS ≥200) was used to evaluate the convergence of the continuous parameters using Tracer v.1.6 (http://tree.bio.ed.ac.uk/software/tracer/).

## Results

### The Prevalence of Measles Cases in the Zhoushan Islands, China

**Figure 1:**
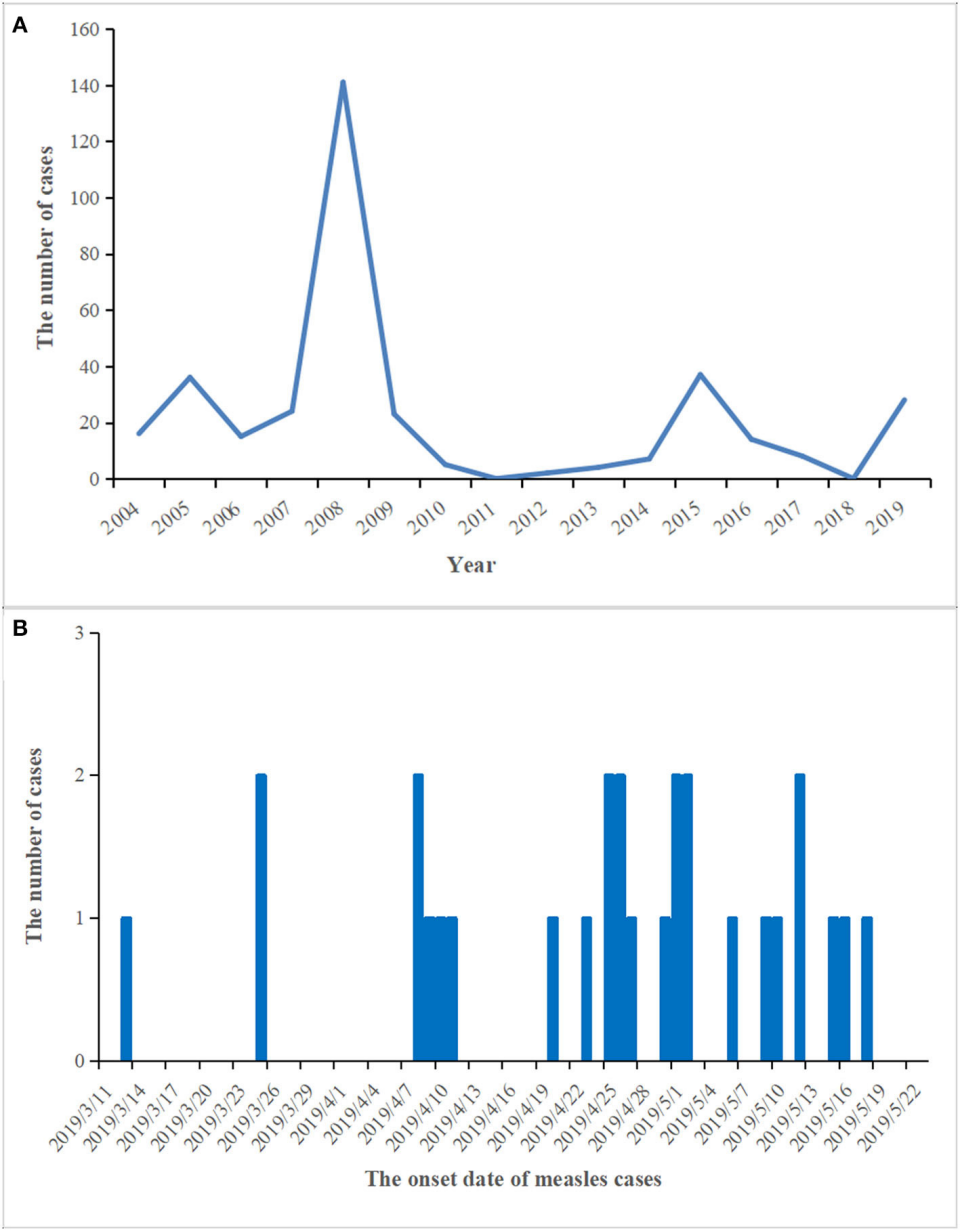
The prevalence and epidemic curve of measles cases in the Zhoushan Islands. **(A)** The reported of measles cases in the Zhoushan Islands during 2004–2019. **(B)** The epidemic curve of measles outbreak in the Zhoushan Islands, 2019.

From 2004 to 2018, the number of reported measles cases ranged from 0 (2011 and 2018) to 141 (2008) in the Zhoushan Islands (Figure 1A). We identified 28 measles cases in the Zhoushan Islands in 2019 (Table 1). The dates of rash onset in the measles cases were between March 13 and May 18, 2019. The incidence rate was 2.4/100,000. Out of the 28 patients, 15 (53.57%) were male and the mean age was 35.5 years (range: 0.5–53 years). All cases had fever and rash, of which 65% had coughs, 35% had conjunctivitis, and 35% had oral mucosal plaques. Four cases were under 6 years of age and none had a history of vaccination. There were no deaths or severe complications among any of the patients. All of the 28 patients were laboratory confirmed as having measles using RT-qPCR; however, six of the 28 swab samples with low viral load (Ct > 36) were negative by RT-PCR, which resulted in no specific genome product available for genotyping. Serologically, only 22 of the 28 patients were measles IgM positive, though all serum samples were collected quickly within 24 h of visiting the hospital (Table 1).

**Table 1:** The patient characteristics and measles virus strains in the Zhoushan Islands, China, 2019.

**Case No.**	**Date of onset**	**Age (Year)**	**Gender**	**Region**	**Occupation**	**Vaccination history**	**Serological test (IgM)**	**Accession number/strain name**
1	13/03/2019	39	Male	Putuo	Fisherman	Unknown	+	MT738528/MVs/Zhoushan.CHN/09.19[D8]
2	25/03/2019	40	Female	Putuo	Self-employed laborer	Unknown	+	MT738529/MVs/Zhoushan.CHN/12.19/1[D8]
3	25/03/2019	51	Female	Putuo	Housework	Unknown	+	MT738530/MVs/Zhoushan.CHN/12.19/2[D8]
4	08/04/2019	29	Male	Dinghai	Self-employed laborer	Unknown	+	MT738531/MVs/Zhoushan.CHN/14.19/1[D8]
5	08/04/2019	44	Male	Putuo	Worker	Unknown	–	MT738532/MVs/Zhoushan.CHN/14.19/2[D8]
6	09/04/2019	36	Female	Dinghai	Public servant	Unknown	+	MT738533/MVs/Zhoushan.CHN/14.19/3[D8]
7	10/04/2019	48	Female	Putuo	Catering industry	Unknown	+	MT738534/MVs/Zhoushan.CHN/14.19/4[D8]
8	11/04/2019	37	Male	Putuo	Business	Unknown	+	MT738535/MVs/Zhoushan.CHN/14.19/5[D8]
9	20/04/2019	29	Female	Dinghai	Teacher	Unknown	+	MT738536/MVs/Zhoushan.CHN/15.19[D8]
10	23/04/2019	8	Female	Dinghai	Student	Unknown	+	–
11	25/04/2019	39	Male	Putuo	Sailor	Unknown	+	–
12	25/04/2019	28	Female	Putuo	Business	Unknown	+	MT738537/MVs/Zhoushan.CHN/16.19/1[D8]
13	26/04/2019	1	Male	Putuo	N/A	None	+	MT738538/MVs/Zhoushan.CHN/16.19/2[D8]
14	26/04/2019	0.5	Male	Putuo	Scattered living	None	+	MT738539/MVs/Zhoushan.CHN/16.19/3[D8]
15	27/4/2019	50	Female	Putuo	Teacher	Unknown	+	–
16	30/04/2019	39	Male	Putuo	Self-employed laborer	Unknown	–	MT738540/MVs/Zhoushan.CHN/17.19/1[D8]
17	01/05/2019	35	Female	Putuo	Medical staff	Unknown	–	MT738541/MVs/Zhoushan.CHN/17.19/2[D8]
18	01/05/2019	58	Male	Putuo	Worker	Unknown	+	MT738542/MVs/Zhoushan.CHN/17.19/3[D8]
19	02/05/2019	49	Male	Putuo	Businessman	Unknown	–	–
20	02/05/2019	35	Male	Putuo	Catering industry	Unknown	+	MT738543/MVs/Zhoushan.CHN/17.19/4[D8]
21	06/05/2019	50	Male	Putuo	Self-employed laborer	Unknown	+	MT738544/MVs/Zhoushan.CHN/18.19/1[D8]
22	09/05/2019	51	Female	Putuo	Housework	Unknown	–	MT738545/MVs/Zhoushan.CHN/18.19/2[D8]
23	10/05/2019	53	Female	Putuo	Tourist	None	+	MT738546/MVs/Zhoushan.CHN/18.19/3[D8]
24	12/05/2019	0.5	Male	Putuo	Scattered living	None	–	MT738547/MVs/Zhoushan.CHN/18.19/4[D8]
25	12/05/2019	49	Male	Putuo	Driver	Unknown	+	MT738548/MVs/Zhoushan.CHN/18.19/5[D8]
26	15/05/2019	3	Male	Putuo	Child care worker	None	+	MT738549/MVs/Zhoushan.CHN/19.19/1[D8]
27	16/05/2019	47	Female	Putuo	Public servant	Unknown	+	–
28	18/05/2019	45	Female	Putuo	Worker	Unknown	+	–

+/− *+ represents positive for IgM test while − represents negative*.

### Epidemiological Investigation of the Outbreak

**Figure 2:**
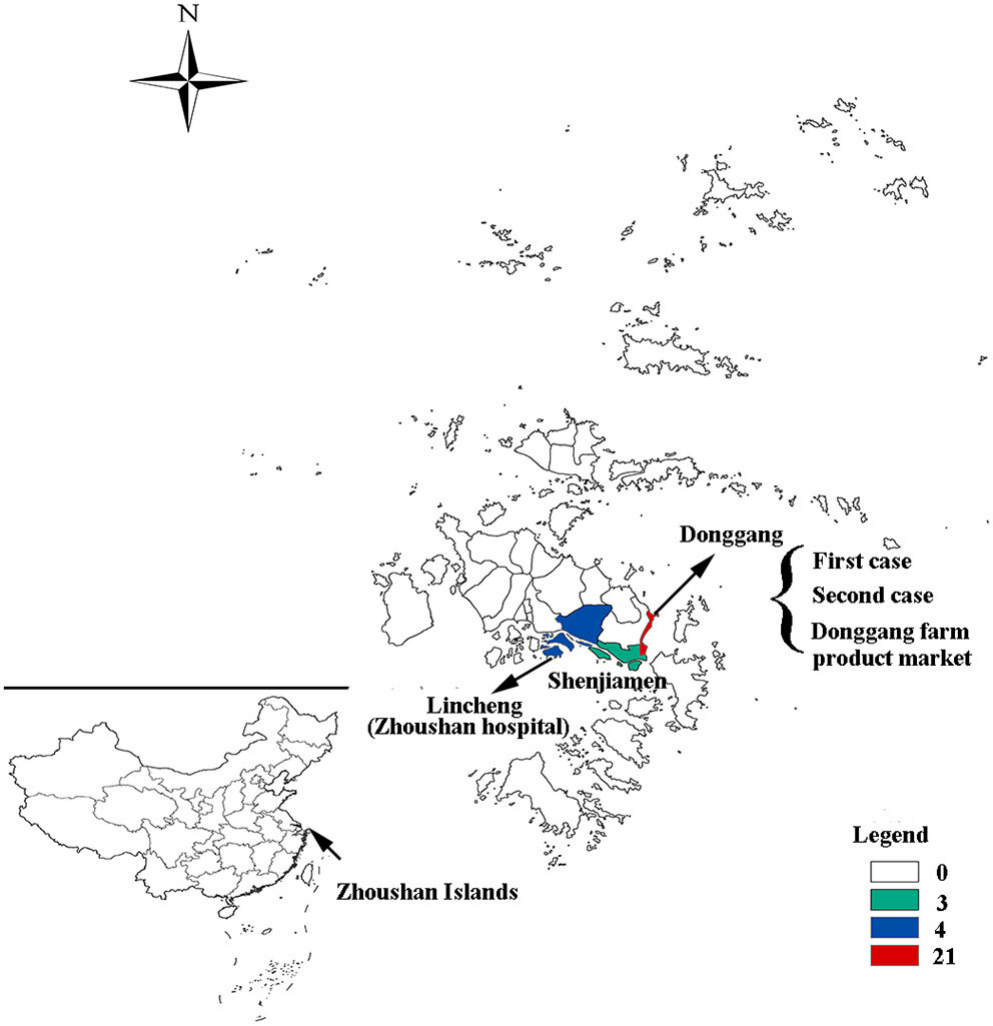
The geographical distribution of measles cases in the Zhoushan Islands. The map showing the geographical distribution of measles cases in the Zhoushan Islands. The colors represent the number of cases in each street in which 21 were in the Donggang street, four were in Lincheng street, and three were in the Shenjiamen street. The first and second cases and Donggang Farm Product Market were located in the Donggang street and Zhoushan hospital was located in the Lincheng street.

Out of the 28 cases, 24 were reported in the Putuo region (Donggang: 21, Shenjiamen: 3), and four in the Dinghai region (Lincheng: 4). The first case was from the Ukraine, who entered China on March 8, 2019 by air directly from the Ukraine to Shanghai Pudong Airport. He arrived in the Zhoushan Islands (Donggang, Putuo region) for a job as a fisherman and then went on a fishing boat on March 9, 2019. He had no history of traveling in other countries 30 days before coming to China. The patient presented with fever and was sent to Zhoushan hospital on March 13, 2019. Two days later (March 15), he was diagnosed with measles infection by RT-qPCR and transferred to a designated hospital for infectious diseases in the Zhoushan Islands. The second case had rash onset on March 25, 2019, 10 days after she visited Zhoushan hospital on March 15, 2019 due to another illness (cough and fever) when both the first and second patient visited the Fever Clinic in Zhoushan hospital. After visiting Zhoushan hospital, the second patient as a self-employed worker went back to work in the Donggang Farm Product Market which is located on the Donggang street in the Putuo region (Figure 2). She was confirmed as having measles by RT-qPCR on March 28 then isolated from March 29, 2019. Her main contacts were her family members, her husband, daughter [16 years old, previously vaccinated with the measles-contain vaccine (*MCV*)], staff at the Market, and customers. The third case, who also had rash onset on March 25, 2019, lived in the same residential area as case 2. Both had contact with the Donggang Market prior to their rash onset. Most of following cases (19/26) were clustered in the Donggang street which is close to the Donggang Farm Product Market (Figure 2). The epidemic curve shows sustained community transmission from March to May (Figure 1B).

### Phylogenetic Analysis Based on the N Gene of Measles Viruses Detected

**Figure 3:**
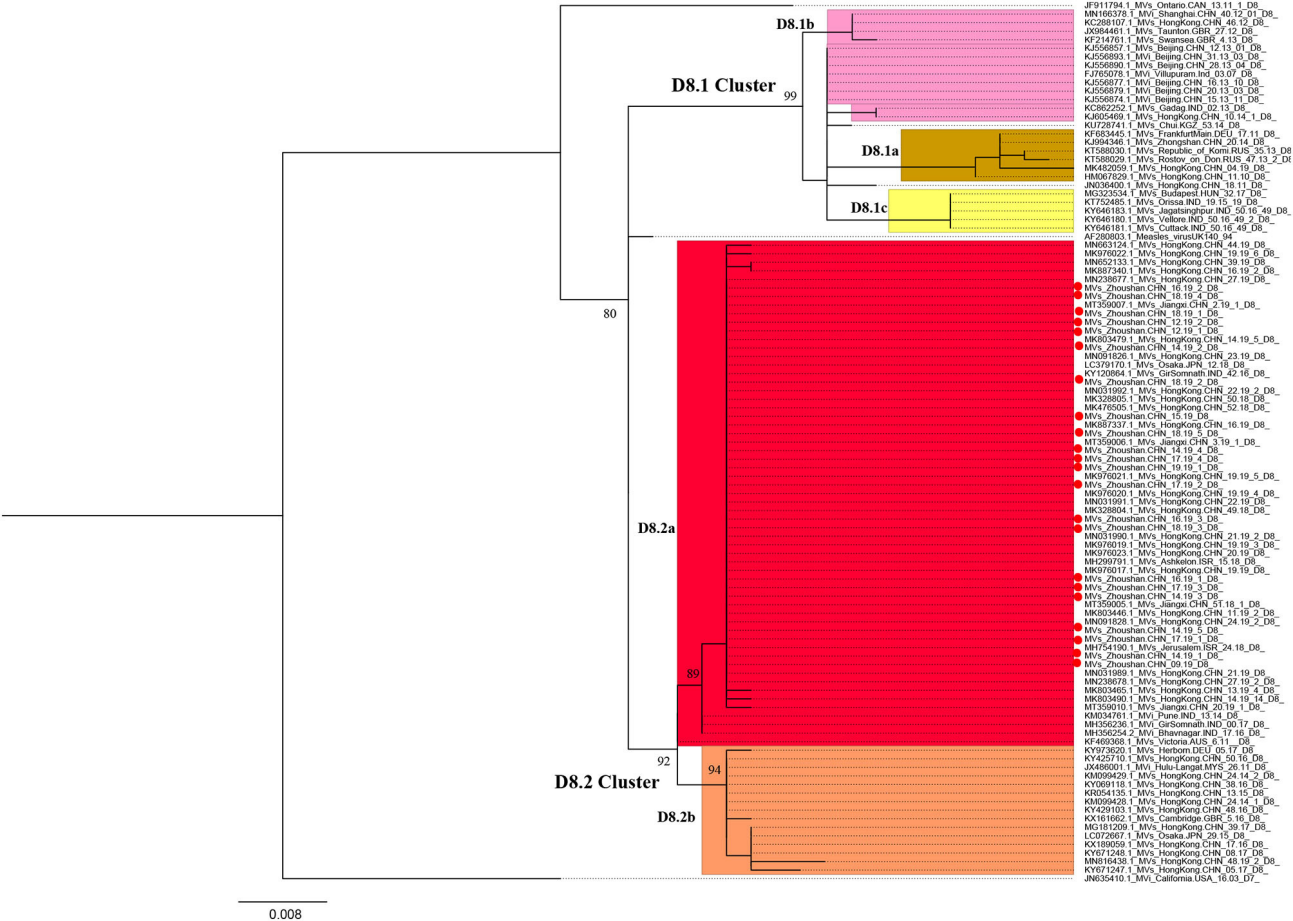
Maximum-likelihood phylogenetic tree of D8 genotype strains based on the 450 bp fragment of the N gene sequences. ML phylogenetic tree showing the genetic relationship among MeV isolates. Strains isolated in this outbreak are represented in red dots. Bootstrap values (>70%) are shown at the corresponding branches and the scale bar is given in substitutions/site.

We obtained the 450 bp fragments of the N gene from 22 of the 28 measles cases and a complete genome from the first case. All sequences are available in GenBank under accession numbers MT738528–MT738550. The phylogenetic analysis was performed based on 103 MeV strains including the 22 strains from the Zhoushan Islands in 2019, a WHO D8 reference strain, 22 WHO-named strains, 49 D8 strains detected in China, and 10 strains which had the closest proximity to the Zhoushan Islands in the initial blast analysis. The measles virus D8 genotype was classified into two groups, D8.1 cluster and D8.2 cluster (25). The D8.1 cluster was further divided into three sub-clusters (D8.1a, D8.1b, and D8.1c) and the D8.2 cluster was divided into two further sub-clusters (D8.2a and D8.2b). The 22 measles virus strains of the Zhoushan Islands were identical and shared 100% identities with KY120864/MVs/GirSomnath.IND/42.16/[D8] based on the N450 region and belonged to genotype D8 (Figure 3).

### Maximum-Likelihood and Time-Scaled Phylogenetic Analysis of the Complete Genome of Measles Virus D8 Strain

**Figure 4:**
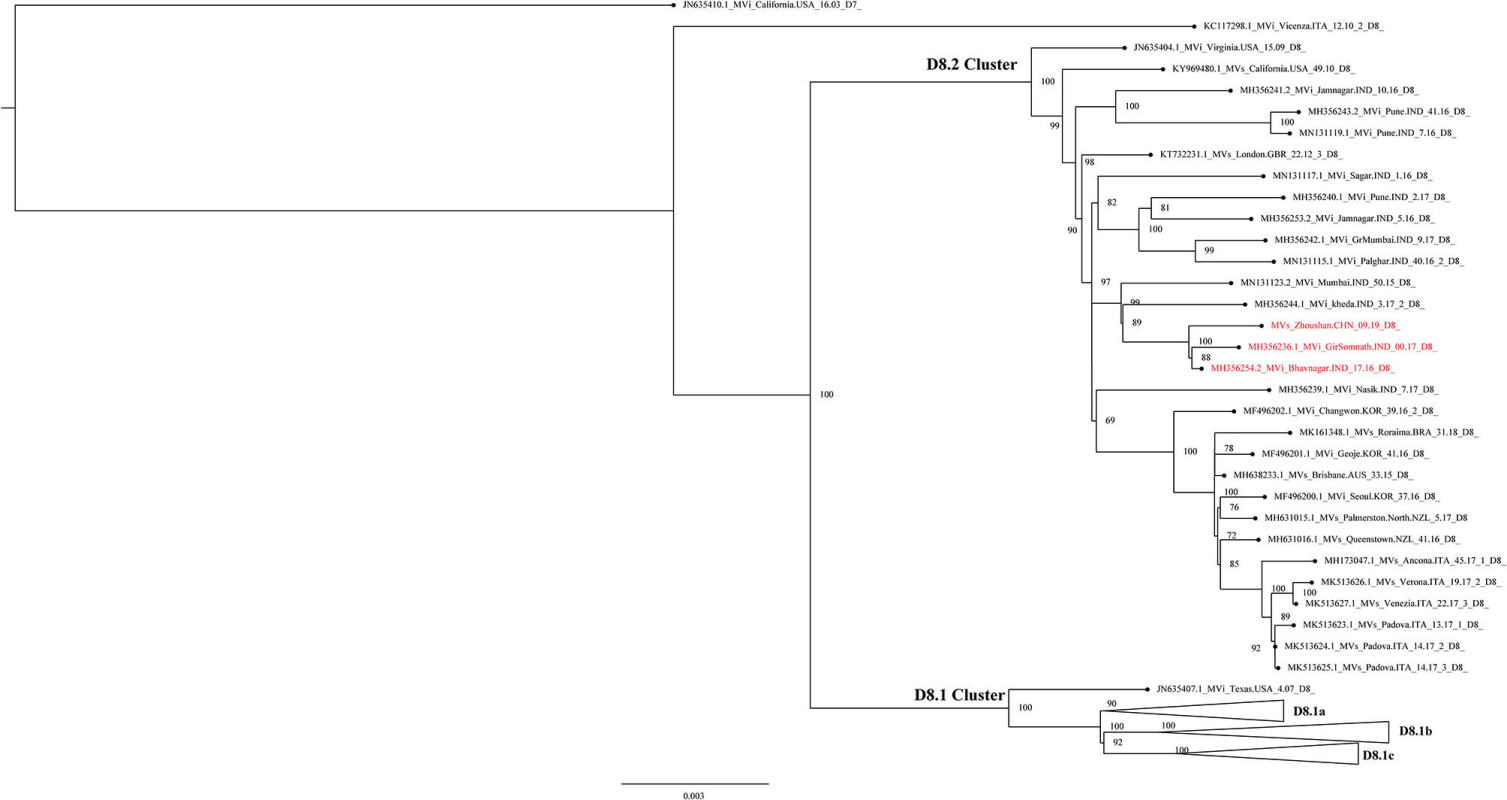
Maximum-likelihood phylogenetic tree of D8 genotype strains based on the complete genome. ML phylogenetic tree showing the genetic relationship among MeV isolates. The triangle represents the D8.1 sub-cluster. The black dot represents each Mev sequence. The scale bar is given in substitutions/site.

**Figure 5:**
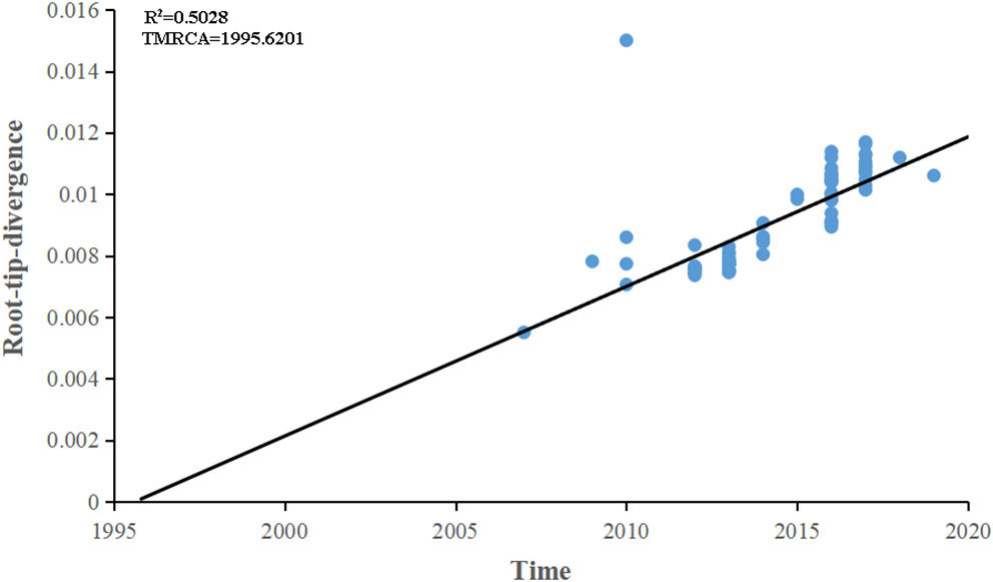
Root-to-tip regression of a maximum-likelihood (ML) phylogenetic tree of complete genome of D8 genotype strains. The correlation between genetic divergence and sampling time was calculated by *R*^2^. The time of the most recent common ancestor was designated by the X intercept.

**Figure 6:**
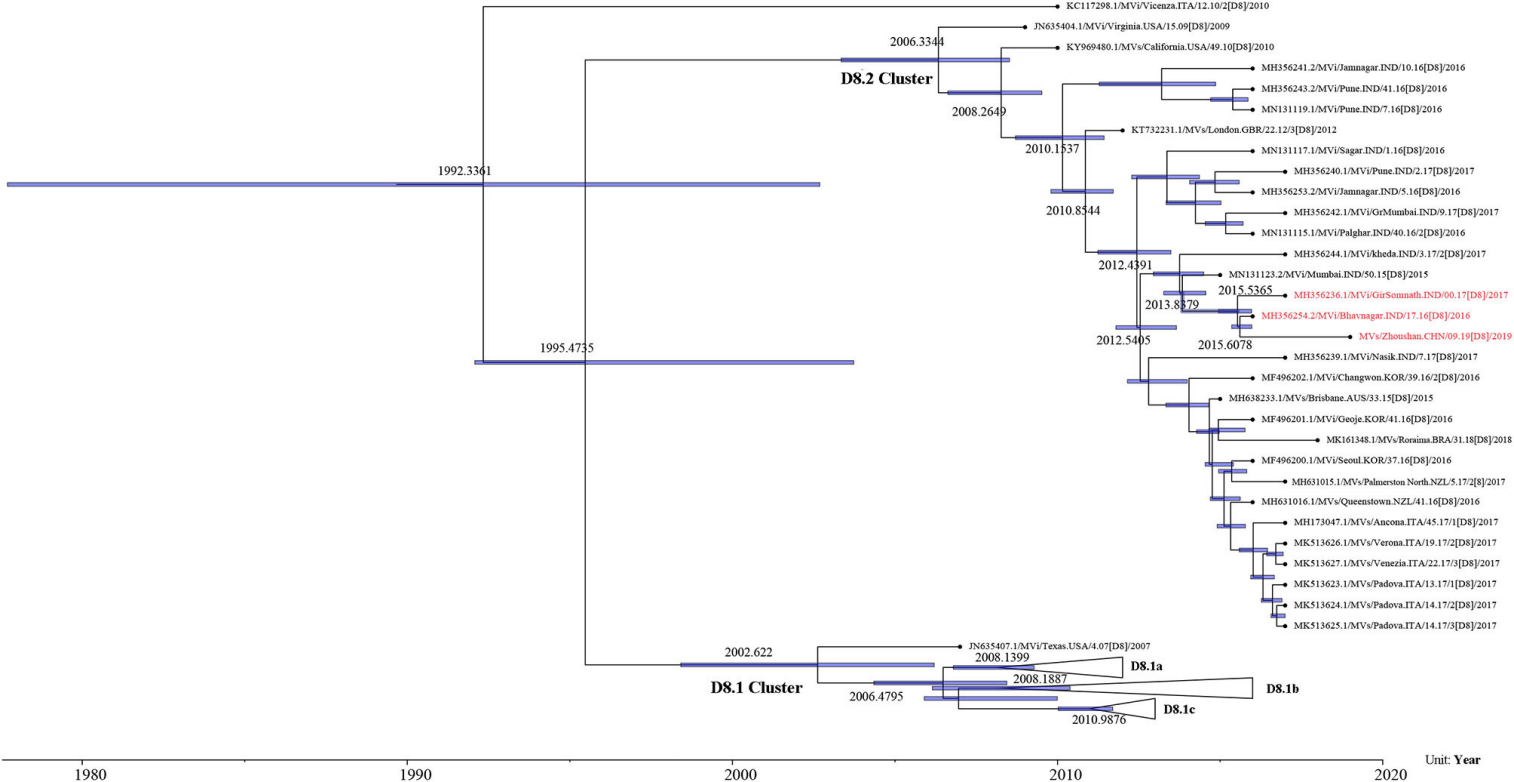
Time-scaled phylogenetic tree based on the complete genome of D8 genotype strains detected from 2009 to 2019. Time-scaled phylogenetic tree showing the evolutionary relationships and timescale of MeV isolates. Branch lengths are scaled according to time. Purple node bars show the 95% credibility intervals of node-age estimates.

The maximum-likelihood phylogenetic analysis of the complete genome obtained from the first patient showed that the MeV strain was clustered with MVi/Bhavnagar.IND/17.16[D8] and MVi/GirSomnath.IND/00.17[D8] with 100 bootstrap values (Figure 4) and shared 99.83 and 99.75% identities, respectively. The dataset exhibited a positive correlation between genetic divergence and sampling time (*R*^2^ = 0.5028) with which the presence of the temporal structure in the sequence data allowed us to proceed with Bayesian molecular dating analyses in BEAST (Figure 5). The complete genome of 80 D8 strains isolated from 2009 to 2019 were collected to construct a time-scaled phylogenetic tree which indicated that measles virus D8 evolved at a rate of 6.91 × 10^−4^ (95% HPD: 5.64–7.98 × 10^−4^) nucleotide substitutions/site/year. The TMRCA for the D8 genotype was estimated to be 1992 (95% HPD interval: 1978–2003) and TMRCA for strains imported to the Zhoushan Islands was estimated to be 2015.61 (95% HPD interval: 2015.36–2015.97) (Figure 6).

## Discussion

In this study, we described a measles outbreak involving 28 cases in the Zhoushan Islands in 2019 where the measles D8 virus was first detected. Apart from this outbreak, no other measles cases were reported in the Zhoushan Islands in 2019. There were no cases reported in 2011 and 2018. The latest measles case was confirmed in 2017 and genotype H1 has only so far been detected in the islands. This status might be caused by the natural recession of the population immunity barrier of MeV. Once measles cases are imported, it is more likely to cause outbreaks. Based on the schedule of the first case, a fisherman from the Ukraine, this outbreak might have been subsequently caused by the virus being imported from the Ukraine. There was then a possible nosocomial infection between the first and second case. Due to the occupation of the second case, she had contact with a large number of people each day and the frequent mobility of the population in the farm product market might have resulted in the spread of the measles virus. The majority of the following cases were clustered in the Donggang street which was close to the Donggang Farm Product Market. After case 2 was identified (March 28), her close contacts were tested and traced by Putuo CDC staff and they requested that she self-quarantine. At the same time, all medical units in the Zhoushan Islands were requested to take continual action in seeking possible measles cases based on clinical surveillance through medical records. During April 8 to April 11, five measles cases were diagnosed and all cases were located in the Donggang street. The Putuo CDC defined this event as an outbreak of measles and reported it to the Zhoushan CDC. Shortly after this, emergency vaccination was conducted among the close contacts as well as the people in the area surrounding where the measles cases worked and lived. Between March 15 and May 20, a total of 764 individuals with specific close contact with a measles case were identified and ~2,000 people susceptible to exposure were vaccinated with the MCV.

In this incident, the vaccination history of 85.71% (24/28) of the patients was unknown, however, 22 of the 28 (78.6%) patients were measles IgM positive suggesting primary infection. The negative IgM in the other six patients could be due to either reinfection or a delayed antibody reaction, e.g., case 24 or the assay sensitivity (Table 1). The reasons for the four cases of young unvaccinated patients were their failure to reach vaccination age (case no. 14 and 24, 2/4) and contraindications associated with MCV (case no. 13 and 26, 2/4). WHO guidelines for the elimination of measles suggest that large-scale outbreaks of measles can be avoided if the MCV coverage is more than 90% in the whole population and that at least 95% coverage could eliminate measles entirely (26). The Chinese government launched the National Expanded Program on Immunization *(EPI)* in 2007 that vaccinated with the MCV at 8 and 18 months of age (27). With these efforts, the measles incidence in China decreased dramatically, from 99.4 per million in 2008 when 151 cases were reported in the Zhoushan Islands (Figure 1A) to 4.6 in 2012 when two cases were reported in the Zhoushan Islands (Figure 1A) (28). However, the actual vaccination coverage in China was estimated to be 80–90% (29). According to the surveillance of the antibody level of MeV in the Zhoushan Islands population in 2014. The overall positive rate was 88.56% and the positive rate of protective antibody was 46.59%. Among the 30–49 age group, the positive rate of antibody and protective antibody were both the lowest, with 84.64 and 40.20%, respectively (30). This indicates that the measles vaccine coverage is not enough in the Zhoushan Islands, especially among the 30–49 age group. When an imported case occurs, it easily causes measles virus infections and outbreaks. Recently, nationwide indigenous measles outbreaks and the resurgence of measles in unvaccinated children have been continuously reported (31, 32). Therefore, it is important to deliver two doses of MCV to children in a timely manner through routine immunization to increase and maintain high coverage (≥95%). Frequent monitoring and assessing coverage are also necessary to identify the pockets at risk that need to be strengthened.

The first measles D8 virus strains identified were AF280803.1/Measles/virusUK140/94 in 1994 in the UK (33). At that time, the D8 genotype had also been detected worldwide including in areas such as South Asia, the Middle East, Europe, and Africa (34, 35). Currently, the MeV genotype D8 is endemic in the Indian subcontinent. The genotype of the measles D8 virus has not been identified by WHO, but other research has classified the D8 virus into two genotypes including D8.1 (D8.1a, D8.1b, D8.1c) and D8.2 (D8.2a, D8.2b) (25). The D8 genotype strains are frequently imported from India into Europe, America, and Asia (36). Nowadays, the D8-Victoria lineage (D8.2a) is one of the predominant lineages worldwide (37). In 2018, measles cases tripled across the European region with nearly 83,000 reported. Among those, the Ukraine had more than 54,000 cases, where in 2016, only 31% of 6 year-old children received the second MCV (38). Because of the Ukraine's large pool of unvaccinated or under-vaccinated people, it is at a high risk of measles outbreaks and importation to other regions. In the maximum-likelihood phylogenetic analysis based on the 450 bp N gene of measles, the strains isolated from this outbreak were clustered with KY120864/MVs/GirSomnath.IND/42.16/[D8] and shared 100% identity with the predominant genotype in the Ukraine during 2018–2019 (39). In this study, MeV genotyping was not available for six patients with low viral loads, however, the results of epidemiological investigation and tracking could prove that they were linked as a part of the outbreak, suggesting that the combination of field investigation and laboratory confirmation is very important in countries or regions working toward measles elimination.

The maximum-likelihood and time-scaled phylogenetic analysis of the complete D8 genotype genome pointed out that this predominant strain originated from India in 2015. In a previous study conducted in India, the genotypes D4 and D8 were combined in the dataset instantaneously and the substitution rate of the D4 and D8 genotypes were estimated at (5.142 × 10^−4^ (95% HPD: 2.697–7.978 × 10^−4^) nucleotide substitutions/site/year) (40). In our study, it was the first time the substitution rate of the D8 genotype genome was calculated independently and it was estimated at 6.91 × 10^−4^ (95% HPD: 5.64–7.98 × 10^−4^) nucleotide substitutions/site/year based on 80 strains isolated from 2009 to 2019. This result would be helpful in evaluating the evolution status of the D8 measles genotype. The high substitution rate of the virus genome always indicates that new variants are more likely to develop which may cause large-scale outbreaks when it spreads to humans. This result was similar to previous studies in which the substitution rate of MeV was estimated to be 3.40–9.02 × 10^−4^ substitution/site/year. The substitution rate of MeV is significantly lower and more static than many other RNA viruses which have the ability to undergo rapid genetic change such as norovirus, influenza A virus, HIV-1 virus, and foot-and-mouth disease which evolved at an approximate rate of 10^−3^ substitution/site/year (41, 42). The D8 strains isolated from Shanghai found that maintained glycosylation of the HA gene virus could still be neutralized by the Chinese measles vaccine strain S191 (H1 genotype) (43). In the first D8 genotype outbreak in Zhejiang province, it was also found that emergent vaccination could stop the transmission of the measles D8 virus, indicating that the current Chinese measles vaccine is effective in preventing measles D8 genotype virus infection (18). The molecular and phylogenetic analysis could provide evidence to confirm the pathways of virus transmission and find variants in a timely manner which could warn us to take immediate prevention and control actions.

Nowadays, the importations of measles cases are challenges to the public health system and herd immunity in all countries (44). Each imported case and related case should be proficiently confirmed and promptly isolated. Before and after an imported case is recognized, their close contacts should be rapidly followed-up (quarantining or exclusion). Vaccinating susceptible individuals and using immunoglobulin are effective in blocking measles transmission in exposed susceptible high-risk people (45).

## Conclusion

In this study, we reported on the first measles D8 genotype outbreak in the Zhoushan Islands which was caused by an imported cases from the Ukraine. There was a possible nosocomial infection between the first case and the second case. Then, the second case played an important role in the spread of virus due to their occupation. The strains isolated in the Zhoushan Islands belonged to the D8.2a sub-cluster and were clustered with KY120864/MVs/GirSomnath.IND/42.16/[D8] which was the predominant genotype D8 strain in the Ukraine during 2018–2019. The analysis of the complete D8 genotype genome pointed out that this predominant strain might have originated from India in 2015. The molecular and phylogenetic analysis could provide evidence to confirm the pathways of virus transmission and find variants in a timely manner. Increasing and maintaining the high level of vaccination coverage (≥95%) and an efficient response to imported cases are essential to prevent and control the recurrence and outbreak of measles virus.

## Data Availability Statement

The raw data supporting the conclusions of this article will be made available by the authors, without undue reservation.

## Ethics Statement

Written informed consent was obtained from the individual(s), and minor(s)' legal guardian/next of kin, for the publication of any potentially identifiable images or data included in this article.

## Author Contributions

HW designed the study. CC, HZ, AT, BW, LL, and MW collected the data. CC and HZ analyzed the data. AT, BW, LL, MW, and HW checked the data and results. CC and HZ interpreted the data and wrote the report. HW revised the report from the preliminary draft to submission. All authors have read and approved the manuscript.

## Conflict of Interest

The authors declare that the research was conducted in the absence of any commercial or financial relationships that could be construed as a potential conflict of interest.
